# The Impact of Surface Discontinuities on MEMS Thermal Wind Sensor Accuracy

**DOI:** 10.3390/s23104575

**Published:** 2023-05-09

**Authors:** Almir Talic, Samir Cerimovic, Roman Beigelbeck, Franz Kohl, Thilo Sauter, Franz Keplinger

**Affiliations:** 1 Institute of Computer Technology, Vienna University of Technology, Gußhausstraße 27-29, A-1040 Vienna, Austria; samir.cerimovic@tuwien.ac.at (S.C.); sauter@ict.tuwien.ac.at (T.S.); 2 Department for Integrated Sensor Systems, Danube University Krems, Viktor-Kaplan Straße 2E, A-2700 Winer Neustadt, Austria; roman.beigelbeck@donau-uni.ac.at (R.B.); franz.kohl.0@gmail.com (F.K.); 3 Institute of Sensor and Actuator Systems, Vienna University of Technology, Gußhausstraße 27-29, A-1040 Vienna, Austria; franz.keplinger@tuwien.ac.at

**Keywords:** thermal wind sensor, FEM (Finite Element Method), Wheatstone bridge configuration, flow measurement

## Abstract

A 2D calorimetric flow transducer is used to study distortions of the flow velocity field induced by small surface discontinuities around the chip. The transducer is incorporated into a matching recess of a PCB enabling wire-bonded interconnections to the transducer. The chip mount forms one wall of a rectangular duct. Two shallow recesses at opposite edges of the transducer chip are required for wired interconnections. They distort the flow velocity field inside the duct and deteriorate the flow setting precision. In-depth 3D-FEM analyses of the setup revealed that both the local flow direction as well as the surface-near distribution of the flow velocity magnitude deviate significantly from the ideal guided flow case. With a temporary leveling of the indentations, the impact of the surface imperfections could be largely suppressed. Including a yaw setting uncertainty of about ±0.5°, a peak-to-peak deviation of 3.8° of the transducer output from the intended flow direction was achieved with a mean flow velocity of 5 m/s in the duct corresponding to a shear rate of 2.4·104 s^−1^ at the chip surface. In view of the practical compromises, the measured deviation compares well with the peak-to-peak value of 1.74° predicted by previous simulations.

## 1. Introduction

The detailed knowledge of the flow of gaseous or liquid media along solid surfaces is a prerequisite for efficient improvements of any devices that have to move through such media. The vast variety of technical applications comprise aircraft and ship design as well as the lubrication of relative motions. Wind sensors for the environment as well as wind tunnel studies are further important examples. Therefore, many micromachined 2D flow sensors of the calorimetric type have been reported in the literature that may be capable of accomplishing such tasks. They can be categorized by the employed temperature sensing technology. Thermoelectric transduction [1,2,3,4,5,6] and thermoresistive technologies [7,8,9,10,11,12,13] are very frequently used. Several excellent review articles are available on various aspects of 2D micromachined flow transducers [14,15,16,17]. Two-dimensional micromachined transducers can help to locally resolve fluid–body interaction in the case of relative motion. Their quick response makes them best suited to track high-dynamic air motions typical for wind sensor applications. Previous simulations revealed that specific transducer designs promise extremely good 2D performance [18,19,20].

As 2D wind sensors are frequently characterized in turbulent flow, the reported specifications are often uncertain. In particular, the dependence of their directional accuracy on the magnitude of the flow velocity is seldom investigated in depth. The work described herein aims at an improved characterization of these transducer features and uncovers related experimental pitfalls.

We focus on a specific transducer design that promises excellent specifications. Previous 3D FEM simulations of the device predicted a moderate deviation of 3° peak to peak for a surface-normal gradient of the tangential velocity of 6.33·10^4^ s^−1^ at the transducer membrane corresponding to a mean guided flow velocity of 10 m/s in the rectangular channel measuring 12 mm in width and 1 mm in height [21].

## 2. Materials and Methods

### 2.1. Sensor Design

In a recent paper, we investigated a large number of layouts of membrane-based micromachined calorimetric flow transducers capable of 2D flow transduction [21]. The common features are resistive thin film devices for heat generation and temperature sensing, all embedded in a thin membrane that is exposed to tangentially flowing fluids. The modeling results of a couple of designs exhibited extraordinary performance in terms of direction-independent flow magnitude transduction and the precision flow direction measurements. Preliminary experimental investigations of a specific transducer design supported the high expectations but only for low flow magnitudes [21].

**Figure 1:**
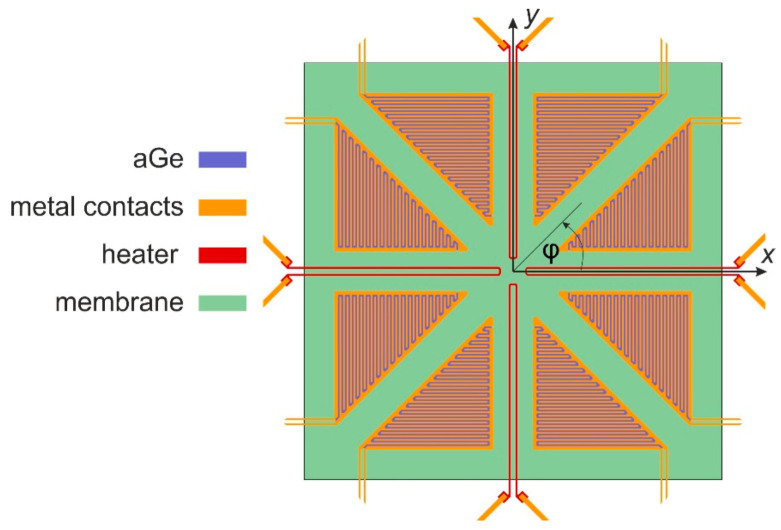
Layout of the sensor membrane, which is supported by a silicon frame, consisting of thin-film devices for heat generation (four U-shaped traces) and temperature sensing (eight triangular structures). The indicated square membrane has a side length of 1.2 mm. The layout results from an iterative attempt to optimize 2D flow transduction with FEM analyses.

The same design is selected for the current experimental study that is devoted to an in-depth experimental investigation of this device. The essential details of the transducer membrane are depicted in Figure 1. Four U-shaped thin-film chromium (Cr) resistors serve for heat generation, while eight thermistors enable distributed temperature sensing. They consist of a triangular amorphous germanium (aGe) layer that is contacted by a pair of comb-shaped metal contacts. The chosen contact geometry (meander-shaped interconnecting leads made of gold) in combination with the high resistivity of the thin aGe layer form the thin-film thermistors. Table 1 gives an overview of the device dimensions. For more details about the material and device layers, refer to [21,22,23].

Due to the high temperature coefficient of resistivity (α = –2%/K) of aGe, this thermistor design enables distributed sensing of locally varying excess temperatures. The sensitivity of different thermal flow sensor designs can only be reasonably compared if they exhibit a similar geometry and layout. If these assumptions are met, the sensitivity of the devices is basically determined by the TCRs of their utilized temperature sensors, which is, in the case of aGe, about five times higher than those of their metal-based counterparts (e.g., platinum) [24].

### 2.2. FEM Studies of Transducer Performance

A sufficiently accurate analytical model is not possible because of the complicated shape of surface discontinuities as well as their complex repercussions on the boundary layer of the flow. Therefore, the focus was only on FEM modelling.

There are several research topics that require different FEM attempts to be accomplished. First, an FEM study enables the prediction of the transduction properties of the device layout, which is essential for device optimization. Second, modeling of the influence of small irregularities at the inner surfaces of the actual flow duct on the flow velocity field in the duct as suggested by experimental findings is carried out. Finally, a conjugated heat transfer study is required to define the velocity range where guided flow measurements enable a reasonable estimate of wind sensing performance of the investigated device. These three models are briefly discussed below.

**Figure 2:**
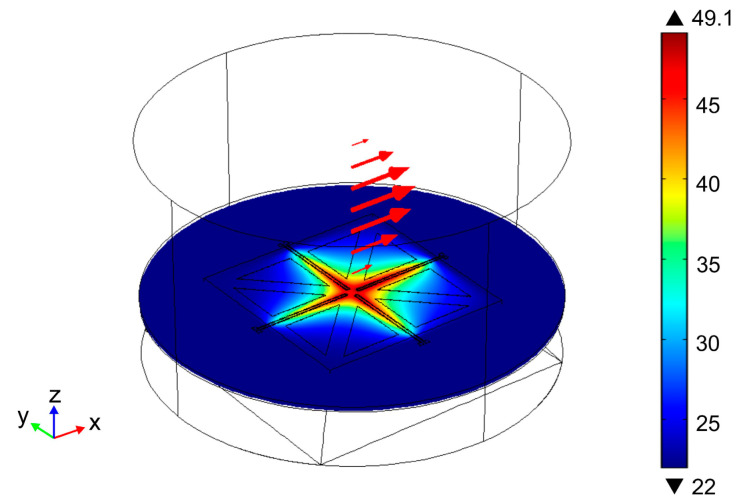
Three-dimensional model for the simulation of the directional characteristic of the investigated transducer. It consists of a cylindrical cutout of the experimental assembly. The colors illustrate the temperature distribution (°C, RT = 22 °C) immediately beneath the surfaces of the device. The fluid domain featuring a height of 1 mm is indicated as transparent wire frame cylinder above the device. As schematically indicated (red arrows), a parabolic velocity distribution with *U*_mean_ = 1 m/s and yaw φ = 0 is applied.

We conducted comprehensive FEM studies of the conjugated heat transfer between a 3D flow velocity field that is partially bound by a quasi-2D array of membrane-borne thin-film resistors acting as heat sources or temperature transducers. Figure 2 depicts a schematic picture of the sensor and the simulation results. Generally speaking, the essential dimensions of the investigated device were implemented into the transducer model. Only a few simplifying measures were taken. The silicon frame is cut into a circular outline of 2.4 mm diameter. To include the comb-shaped metal contacts at the surface of the aGe thermistor film, the triangular temperature sensing elements are treated as a single effective material with anisotropic heat conduction parameters. The four U-shaped heater strips were approximated by a single block featuring the same thickness as the adjacent thermistors. The thickness of the membrane and the therein embedded elements was enlarged by a factor *a* = 20 to reduce meshing complexity. Of course, the material properties were appropriately scaled, as well. Due to the high thermal conductivity of Si, the complete frame remains very close to room temperature. Therefore, the simulation model can be tightly cut around the relevant membrane area specifying an RT (room temperature) boundary condition at the respective bounds of the model. As only moderate excess temperatures are of interest, the temperature dependencies of the fluid parameters can be ignored. Perpendicular to the flow direction, a fixed-velocity distribution can be imposed in correspondence with the flow channel profile. The *x*- and *y*-components of the velocity are computed from the sine and cosine function of the yaw φ. For more details about the FEM model implementation, operating modes, and quality, refer to [21,22,23]. 

### 2.3. Transduction

In this work, the sensor is operated only in the calorimetric mode. This measurement principle evaluates the temperature difference of temperature sensors, located around a separate heater structure, which serves as a heat source [14,17,18]. It can also be operated as some kind of hot-film sensor, where the heater is switched off and the self-heating effect of the temperature sensors is utilized as a heat source. However, in this operating mode, the output signal is strongly dependent on the variation in the ambient temperature, which must be detected during the measurement in order to correct the output signal [22].

**Figure 3:**
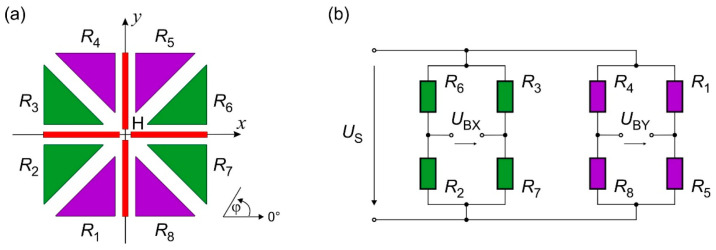
(**a**) Schematic layout of the wind sensor comprising a central heater structure (H) and eight aGe-thermistors (*R*_1_–*R*_8_). (**b**) Interconnecting scheme of the aGe-thermistors to two full bridge configurations supplied by a constant voltage *U*_S_. The bridge voltages *U*_BX_ and *U*_BY_ correspond to the *x*- and *y*-directions of the flow velocity vector.

Figure 3a shows the schematic layout of the sensor. A thin-film heater structure (H) is placed at the center of a membrane surrounded by eight thermistors (*R*_1_–*R*_8_) made of aGe. The temperature profile induced by the heater depends on the flow direction and velocity, so with a thermistor array arranged as depicted in Figure 3a, it is possible to measure both components of the flow velocity (*v_x_* and *v_y_*). Thus, information about the flow direction and the magnitude of the flow velocity can be obtained simultaneously [21].

The eight aGe-thermistors are connected in a double full bridge configuration, as illustrated in Figure 1b. The bridge voltage that corresponds to the *x*-direction of the flow velocity equals

(1) $$U_{\mathrm{BX}}=U_{S}\times \left(R_{2}R_{3}-R_{6}R_{7}\right)/\left(R_{6}+R_{2}\right)\left(R_{3}+R_{7}\right)$$

A similar equation can be obtained for the voltage, which corresponds to the *y*-direction of the flow velocity vector:

(2) $$U_{\mathrm{BY}}=U_{S}\times \left(R_{1}R_{8}-R_{4}R_{5}\right)/\left(R_{4}+R_{8}\right)\left(R_{1}+R_{5}\right)$$

In Equations (1) and (2), *U*_S_ denotes the constant supply voltage of the Wheatstone bridges (cf. Figure 3).

Without a flow, the heater generates a rotationally symmetric temperature distribution on the sensor membrane. Consequently, all thermistors detect approximately the same temperature and both output signals (*U*_BX_ and *U*_BY_) tend to zero. Small differences in the measured temperatures are the reason why *U*_BX_ and *U*_BY_ typically feature a low offset. They arise from manufacturing tolerances of the aGe-thermistors as well as the imperfect symmetry of the real layout. However, this is a systematic error that can be removed during subsequent data processing [22].

The tangential fluid flow over the membrane distorts the initial symmetry of the induced temperature field. Assuming the flow is in the positive *x*-direction (i.e., φ = 0°), the upstream thermistor pair (*R*_2_ and *R*_3_) is cooled down more intensively than its downstream counterpart (*R*_6_ and *R*_7_). An aGe-thermistor features a negative temperature coefficient of resistivity, i.e., its resistance increases with decreasing temperature [22]. Hence, the difference in the numerator of Equation (1) reaches its maximal value if the thermistors are connected as depicted in Figure 3b. On the other hand, for the flow in the positive *y*-direction (i.e., φ = 90°), the downstream thermistors (*R*_3_ and *R*_6_) and the upstream ones (*R*_2_ and *R*_7_) measure the same temperature, respectively. In this case *U*_BX_ equals zero, while at the same time, the voltage *U*_BY_ reaches its maximum. Moreover, as the sensor features a central symmetric layout, the directional characteristic of these two output quantities is approximately sinusoidal. Therefore, the angle of the flow direction with respect to the positive *x*-direction can be calculated by

(3) $$\Psi =\arctan\left(\frac{U_{\mathrm{BY}}}{U_{\mathrm{BX}}}\right),$$

whereas the flow velocity magnitude is a function of the modulus of the bridge voltages

(4) $$\left|U_{B}\right|=\sqrt{U_{\mathrm{BX}}{}^{2}+U_{\mathrm{BY}}{}^{2}}$$

The function depends not only on the flow velocity *v*, but also on thermistor properties as well as the supply voltage *U*_S_, and must be obtained experimentally.

The temperature dependence of the aGe-thermistors can be best fitted by an exponential function [25]:

(5) $$R_{i}=R_{0}e^{\alpha (T_{A}+\Delta T_{i})}$$

where index *i* indicates the thermistor position (cf. Figure 3a), whereas α and *R*_0_ are the temperature coefficient of resistivity and the thermistor resistance at the reference temperature of 0 °C, respectively. *T*_A_ is the ambient temperature in °C and Δ*T_i_* denotes the variation in the thermistor temperature depending on the flow direction and velocity. Since all thermistors were fabricated within the same process flow, they feature approximately equal characteristic parameters (α and *R*_0_). Inserting temperature dependence (5) into Equations (1) and (2), we can conclude that in the first approximation, the bridge voltages *U*_BX_ and *U*_BY_ are independent of the ambient temperature *T*_A_. This is the decisive advantage of the double bridge configuration (supplied by a constant voltage) over a conventional calorimetric [26,27] or a hot-film operating mode [28], where variations in the ambient temperature cannot be neglected.

**Figure 4:**
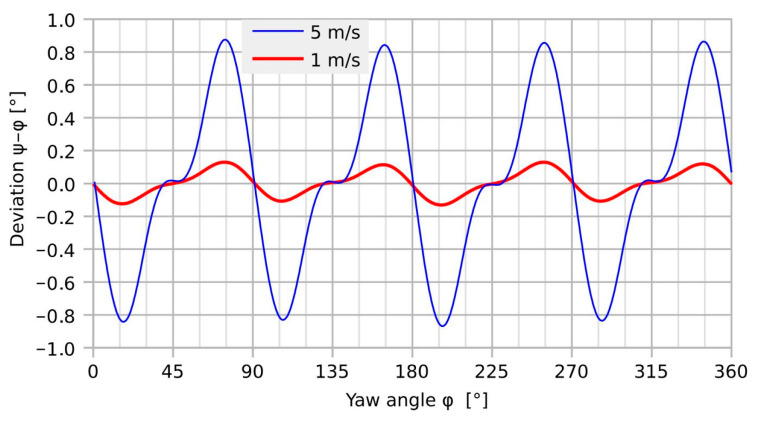
Simulated transducer deviation of the presented device design at *U*_mean_ = 1 and 5 m/s. Obviously, a distinct 90° period of the deviation occurs in contrast to the previous measurement results [21].

Figure 4 displays the simulation results for the transducer deviation as a function of the yaw angle for the device design according to Figure 1.

As suggested by the symmetry of the device design, the simulated deviation exhibits a main period of 90°. However, the details of these deviation characteristics indicate more complex transduction behavior [21]. The minimum error is observed around multiples of 45° because both heat generation and temperature detection are symmetric with respect to the flow direction. In contrast, the lack of that symmetry leads to maximum angular deviations at about a ±15° offset from the *x*- and *y*-axes. Furthermore, a significant increase in the moderate deviation is obtained with increasing flow velocity. The peak-to-peak deviation amounts to 0.26° and 1.74° for *U*_mean_ = 1 and 5 m/s, respectively. The corresponding peak-to-peak variation in the magnitude transduction ranges from 0.13 to 0.41%.

**Figure 5:**
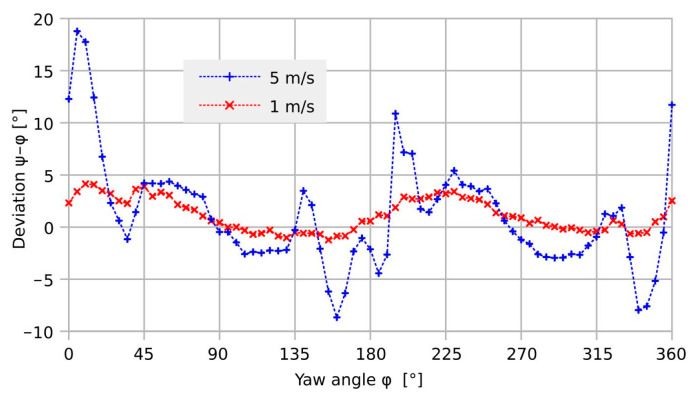
Deviation of the measurement flow direction ψ (computed from *U*_BX_, *U*_BY_ measurements) from the preset yaw angle. A transducer carrying epoxy bumps that protrude into the flow channel was used with mean flow velocities of 1 m/s and 5 m/s.

In contrast to the encouraging simulation results, preliminary measurements [21] yielded a vast peak-to-peak deviation of 27.4° at 5 m/s (see Figure 5). As it is hardly probable that this massive mismatch is caused by the transducer element itself, further research is reasonable to find the ultimate performance of the transducer. 

The tremendous experimental deviation was attributed by intuition to bumps of epoxy resin that are situated at two opposite edges on the surface of the transducer chip. These bumps serve to protect the bonding wires from mechanical damage. However, they locally constrict the regular cross-section of the rectangular flow channel, causing deflection and magnitude irregularities of the flow velocity field near the transducer membrane surface.

Therefore, an improved flow measurement setup was implemented to study the directional characteristic of the transducer element itself in more depth.

### 2.4. Flow Measurement Setup

For the research reported herein, we relinquish any protection measures of bonding wires. That strategy severely restricts the handling of the flow transducers and allows only investigations in a carefully controlled laboratory environment. Each unprotected device was incorporated once into the flow channel and then characterized by a comprehensive measurement series. 

**Figure 6:**
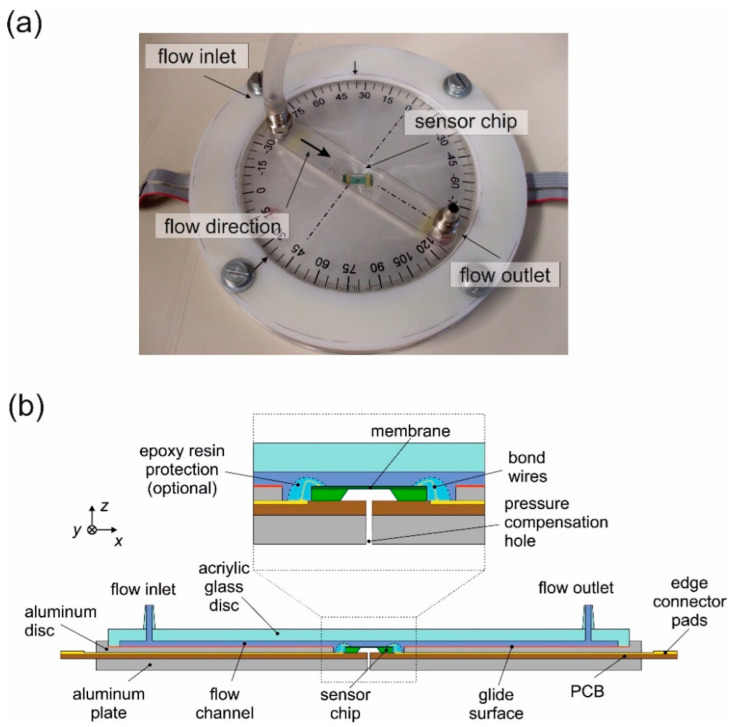
(**a**) Laboratory setup for transduction studies and recording of the flow direction. (**b**) Cross-sectional view of the upper assembly. The cut plane is oriented along the rectangular flow channel. The acrylic glass disk can be rotated against all other parts by 360°.

A special device was developed to characterize the angle dependency [21,22], as shown in Figure 6a. The transducer chip was flush-integrated at the center of a plane aluminum disk that forms the bottom border of a slim rectangular compartment. The radius of the aluminum disk is 8.5 cm. The distance between the inflow and outflow outlets is 7.3 cm. The remaining walls of the compartment are milled into the surface of a transparent PMMA disk forming an indentation of 12 mm width, 1 mm depth, and 85 mm length. The aluminum disk can be rotated against the other compartment walls by 360° around the center of the chip. The sliding interface of these disks was sealed with vacuum grease. 

Adding two feed-throughs to the PMMA disk turns the compartment into a hinged rectangular flow channel. Relative rotation of the disks provides an equivalent rotation of the tangential flow velocity at the transducer surface.

The channel dimensions ensure laminar Hagen–Poiseuille flow throughout the applied range of mean flow velocities up to 5 m/s (Re < 350). Filtered nitrogen was used for the gas flow in the flow channel, where the velocity of the nitrogen was adjusted by a calibrated flow controller. Since the flow channel was sealed, no external flow disturbances are expected. The transducer chip is supported by a PCB that sits in a slot at the backside of the aluminum disk. As the micromachining technology at hand lacks through-wafer vias, wire-bonded interconnections from the chip surface to pads on the PCB carrier are mandatory. Therefore, small rectangular openings in the aluminum disk have to be provided next to the chip to allow for interconnecting wires.

## 3. Results

### 3.1. Flow Characterization Results

#### 3.1.1. Basic Transduction Properties

**Figure 7:**
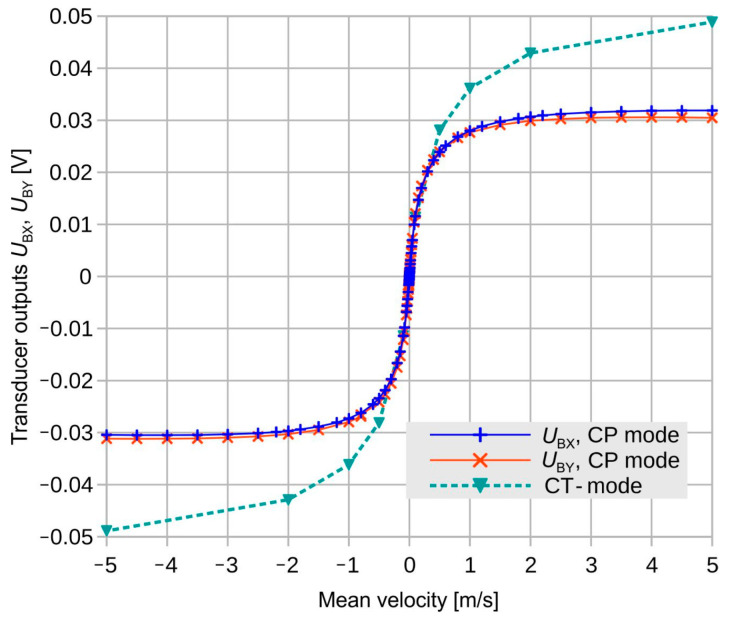
Measured flow magnitude transduction for flow velocities along the *x-* and *y*-design coordinates and a constant heating power of 3 mW (CP operating mode). In addition, a proposed characteristic for constant-temperature operation (CT mode) is indicated.

Using a total heating power of 3 mW, the flow magnitude transduction shown in Figure 7 was measured for velocities along the *x*- as well as the *y*-axis of the transducer design. Zero-flow offset voltages have been subtracted in advance. Because of the dependence of *U*_BX_, *U*_BY_ on the flow magnitude, these signals are coupled in principle. To interpret flow measurements in terms of flow direction and magnitude, both signals have to be evaluated in conjunction.

Obviously, each measured transduction characteristic exhibits a slight deviation from point symmetry. Furthermore, the *U*_BY_ characteristic deviates from its *U*_BX_ counterpart. Among other reasons, a slight misalignment of the transducer chip with respect to the channel boundary, as well as tolerances of the thermistor resistances, can be responsible for this phenomenon.

Occasionally, the dependence of all eight thermistors’ resistances on the flow velocity was recorded as well. From the measured thermistor resistances, the mean membrane excess temperature and an effective thermal resistance at the actual flow rate can be computed according to the imposed heating power. From the thermal resistance, the heating power required for maintaining a constant excess temperature can be calculated. Assuming linear relations between heating power and thermistor temperatures, transducer signals for the constant-temperature operational mode (CT mode) can be computed from the CP (constant power) signals at any specified flow velocity. This procedure was performed for a couple of flow velocities and the estimates for CT transduction are displayed in Figure 7, as well.

For in-depth studies of calorimetric flow transduction, the CP mode is more appropriate since it does not involve possible interferences by an external temperature controller system. The pronounced saturation or even a slight reversal of the CP characteristics at high flow velocities is typical for calorimetric transduction. There, the entire transducer membrane becomes efficiently cooled by the fluid. The constant excess temperature mode of operation (see Figure 7) is a consequence of this. The CT mode is of great advantage when flow velocities should be deduced from transducer signals, i.e., for flow sensing applications. Moreover, the CT servo loop provides further signals featuring a monotonous dependence on the flow velocity.

#### 3.1.2. Directional Characteristic at Low Flow Rates

The mechanism used for flow field rotation was conceived for in-depth investigations of 2D flow transduction properties of the micromachined devices. Epoxide bumps were omitted to avoid any interference of the transducer with the fluid flow. However, further imperfections of the setup that have been considered insignificant cause severe distortion of measurement results. The excellent properties of the investigated device helped to unveil even minor deficiencies in the characterization setup used.

Two lateral extensions of the opening in the bottom wall of the flow channel are required to allow for wired interconnections to the carrier PCB. The PCB at the bottom of these extensions turns them into lateral recesses with a depth of 0.35 mm, i.e., the thickness of the micromachined chip. Intrinsic deficiencies of the transducer and imperfections of the characterization setup become obvious in the measured directional diagrams.

To generate directional diagrams, the rotation angle was incremented by steps of 5° between zero and 90°.

**Figure 8:**
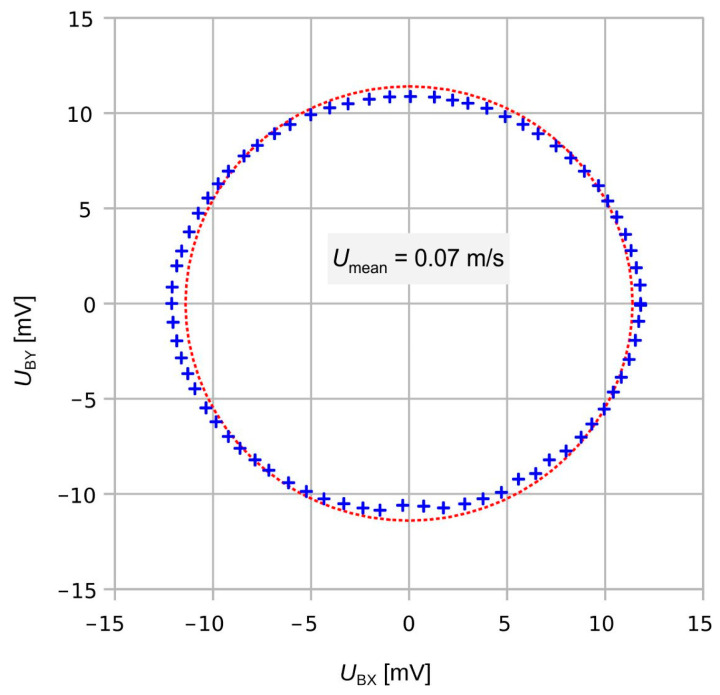
Directional diagram of the transducer output at *U*_mean_ = 0.07 m/s along with its least-square fit circle (red dots). The zero-flow offset of the bridge voltages has been subtracted previously. An anisotropy of about 10% is observed between bridge signals *U*_BX_ and *U*_BY_.

In the region of moderate mean flow velocities (<1 m/s), the raw transducer bridge signals give approximate elliptical directional diagrams, as shown in Figure 8, for example. The *U*_BY_ signal seems down-scaled compared to *U*_BX_. Perhaps that observation may originate from different transduction efficiencies of the two thermistor bridges. Alternatively, the flow velocity across the transducer membrane could be smaller if the flow channel axis aligns with the *y*-coordinate of the transducer structure. 

To support the interpretation of these experimental observations, further modeling studies were required. A 3D laminar flow model was established to investigate the velocity field in a rectangular flow channel featuring local indentations at one boundary. 

**Figure 9:**
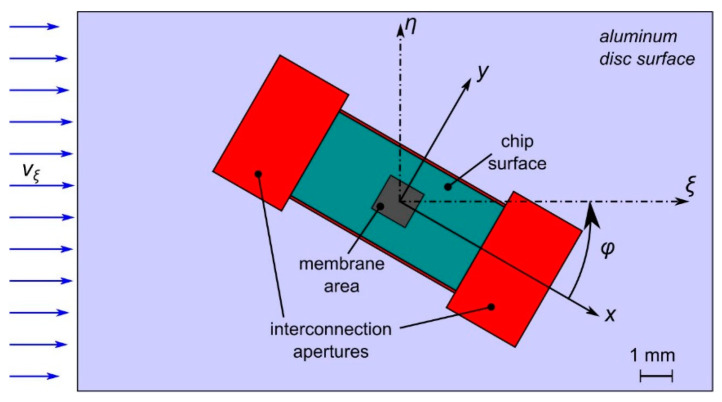
Map of the bottom bound of the flow channel for FEM modeling. The depth of the recesses is color coded: turquoise 50 µm (chip area), red 350 µm (wirebonds and lateral clearances). The yaw angle φ in all diagrams refers to the *x*-axis of the chip layout, while the FEM model keeps the channel axes *ξ*, *η* fixed in order to simplify the definition of the inflow boundary condition.

A 20 mm long section of the flow channel is modeled comprising the actual wall indentations around the transducer chip in the bottom bound of the flow as well as a slightly lowered position of the chip surface (see Figure 9). To save modeling efforts, any rotation of the flow channel is replaced by an equivalent clockwise rotation of the chip assembly. At the flow inlet, a velocity profile

(6) $$v_{\xi }\left(\eta ,\zeta \right)=v_{max}\cdot \left(1-{\left(\frac{2\eta }{w}\right)}^{m}\right)\cdot \left(1-{\left(\frac{2\zeta }{h}\right)}^{2}\right)$$

is specified, where *w* denotes the width of the bottom bound, *h* denotes the height of the flow channel, and *m* = 16.8 follows from the actual aspect ratio of 12 of the cross-section. The Cartesian coordinate *ξ* points along the flow direction, while *η* and *ζ* originate at the center axis of the channel (see Figure 9). Equation (6) is an easy-to-evaluate approximation to the exact theoretical velocity distribution in rectangular ducts given in Section 4 [29]. For the cross-sectional distribution according to Equation (6), the maximum velocity amounts to 1.63 times the mean velocity. 

Indeed, the 3D FEM studies revealed that the required recesses cause flow velocity field distortions that depend on the yaw of the transducer against the channel axis. The simulation model of Figure 9 represents a cutout of 20 mm length of the flow channel of *w* = 12 mm. A velocity distribution according to Equation (6) and *h* = 1 mm is specified at the left model boundary with *U*_mean_ = 5 m/s and *v*_max_ = 1.63∙*U*_mean_. With respect to the bottom channel wall, the two recesses at the short edges of the chip measure 2.5 mm in width, 4.2 mm in length, and 350 µm in depth. Furthermore, the chip surface of 3 mm by 6 mm is lowered by 50 µm. Manufacturing tolerances further require two trenches of 350 µm depth and 100 µm width along the long edge of the transducer chip.

To illustrate the resulting flow velocity field, streamline plots were computed. Furthermore, the surface shear rate at the membrane site was evaluated to aid the interpretation of the experimental observations.

**Figure 10:**
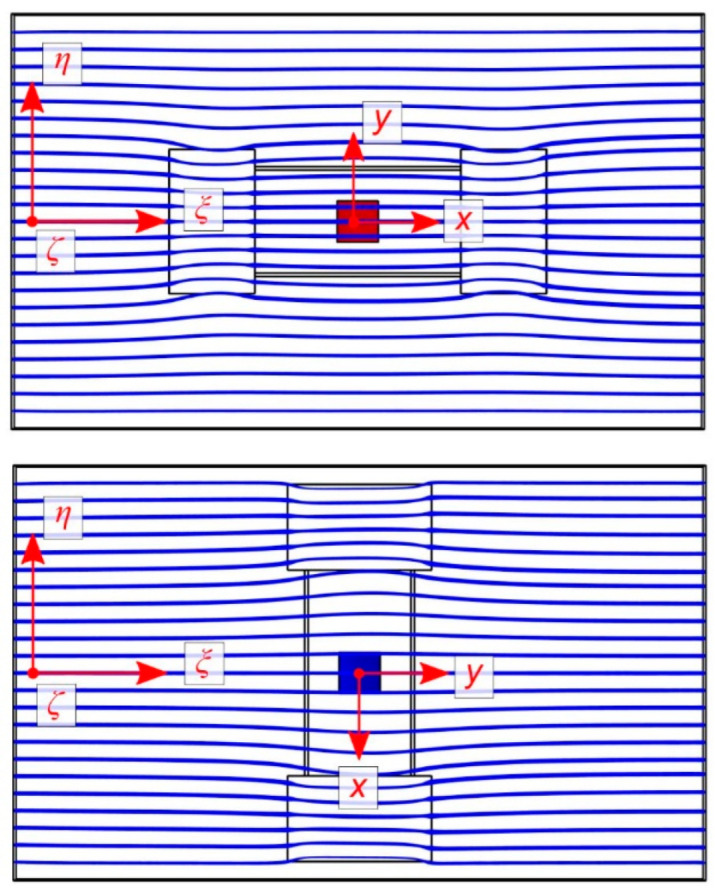
Streamline bending for chip rotations of 0° and 90°. Equidistant starting points are chosen 100 µm above the bottom channel wall. The width of the streamlines encodes the local velocity magnitude. The circumferences of the indentations around the transducer mount are indicated.

Figure 10 illustrates the observed behavior. If the relative rotation directs *v*_ξ_ along the *y*-axis of the device layout, the recesses for the wired interconnections form bypasses for the fluid in collateral position to the transducer membrane. These bypasses attract streamlines of the flow field, which is concomitant with a reduction in the flow velocity at the membrane site. If, however, *v*_ξ_ coincides with the *x*-axis, these recesses are flush with the membrane site. The increase in the channel cross-section occurs at the middle of the channel wall, which slightly attracts the flow field towards the transducer membrane location. 

**Figure 11:**
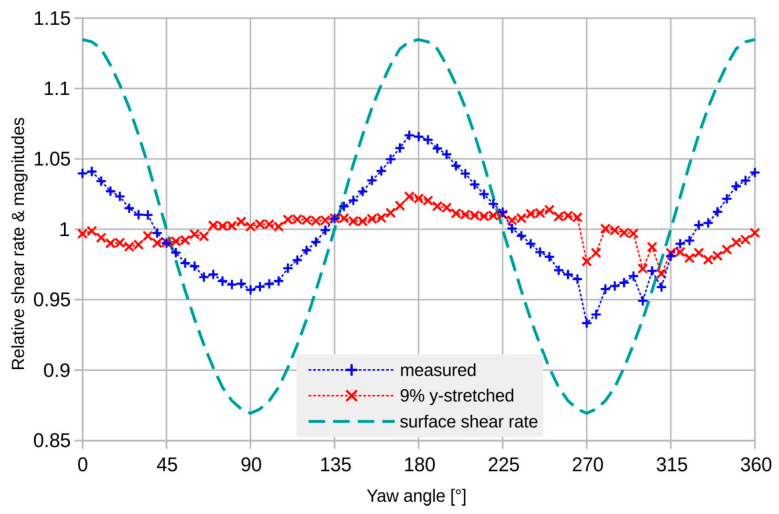
Directional variation in the signal magnitude for *U*_mean_ = 0.1 m/s; as measured (+) and with an arbitrary 9% numerical rectification of the bridge signals (x) to compensate for the influence of bonding recesses. The appropriately normalized variation in the average surface shear rate (--) is also displayed, obtained for the membrane area from simulations.

For intermediate angles, the conditions change gradually between the extreme cases of Figure 10. The angular dependence of the average surface shear rate of the transducer membrane confirms this view (see Figure 11). There is an obvious correlation between the surface shear rate and the flow magnitudes derived from the measured transducer signals.

**Figure 12:**
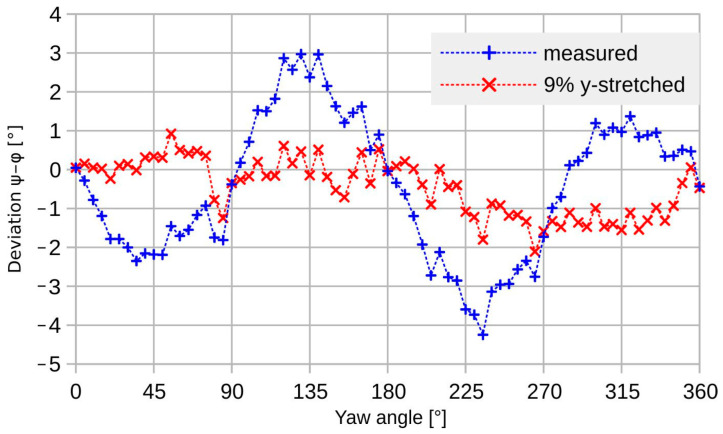
Measured transducer deviation (+) and the improved characteristic after a 9% numerical stretch of the *y*-bridge signal (x). Mean flow velocity 0.1 m/s.

A first-order correction by a single relative scaling of the bridge signals markedly reduces the deviation as well as the directional dependence of the magnitude transduction (see Figure 11 and Figure 12). The applied relative scaling of 1.09 gives low transducer deviation as well as good uniformity of magnitude conversion.

A similar improvement in the measured deviation is achieved based on evaluation of the scaled bridge signals (see Figure 12). 

At low guided flow velocities, simple scaling of the *U*_BY_ signal with respect to *U*_BX_ improves the magnitude uniformity from about 10%_pp_ (peak-to-peak) to less than 3%_pp_ and the deviation from more than 7°_pp_ to about 3°_pp_, both including the random adjustment uncertainty of approximately 1°. Compared to the corresponding simulation results, the corrected peak-to-peak deviation is more than an order of magnitude higher. Furthermore, the measurement result shows no variation that can be ascribed to the transducer design, similar to the characteristics of Figure 4. The measured deviation is very likely a characteristic for the measurement setup and not for the transducer device.

To summarize the observations at low flow velocities, both the magnitude error and the deviation can be significantly improved by simply scaling the component signals of the transducer.

#### 3.1.3. Directional Characteristic at High Flow Rates

**Figure 13:**
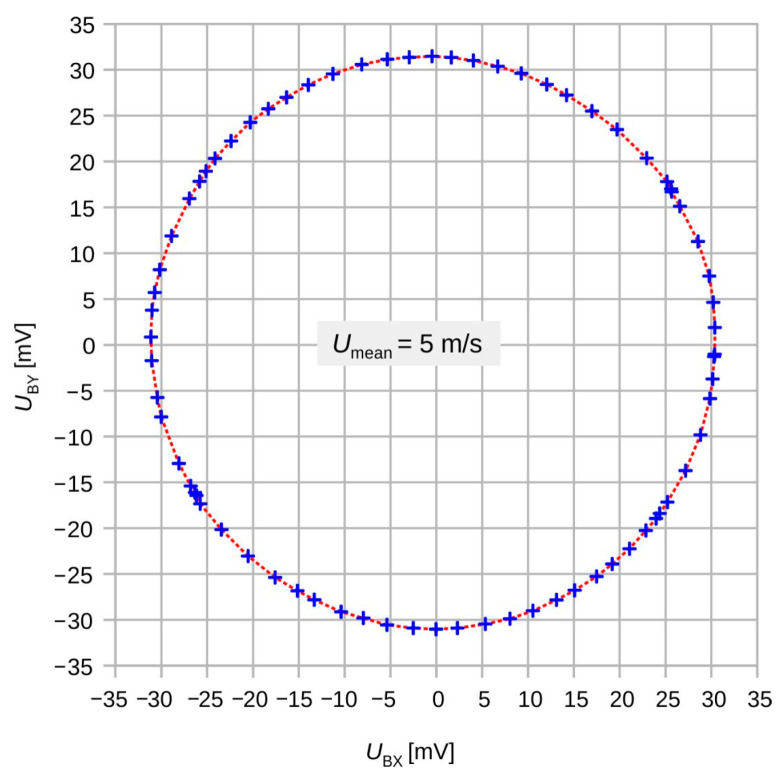
Measured directional diagram of the transducer signals *U*_BX_ vs. *U*_BY_ (zero-flow bridge offsets removed) for *U*_mean_ = 5 m/s and completely open recesses (blue + marks). The excellent match with the least-square circular fit is in contrast with an erratic density of the measurement points (red dots).

At high flow velocities > 1 m/s, the measurements results become rather intriguing. Figure 13 depicts a directional diagram constructed from the offset-corrected transducer signals *U*_BX_ and *U*_BY_. As the flow direction was incremented in fixed steps of 5°, the irregular azimuthal positions of the measurement points signaled a distinct transducer deviation. The nearly perfect circular shape of the directional diagram has two premises. First, it is a consequence of the saturation of the transduction characteristic at high flow velocities (see Figure 7) that levels out any flow magnitude dependence. Second, a unique conversion efficiency for both velocity components applies independently of the actual values of the velocity components. This observation holds for all investigated velocity magnitudes. Hence, the flow transduction of each thermistor bridge is not solely dependent on the respective velocity component but entirely controlled by the flow magnitude. 

**Figure 14:**
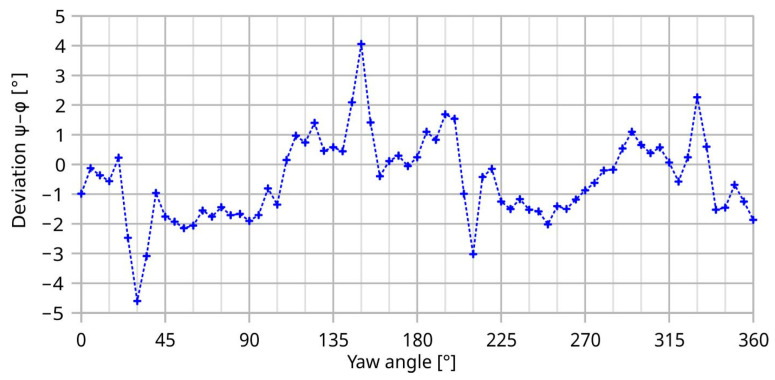
Measured transducer deviation at *U*_mean_ = 5 m/s.

For 5 m/s, we measured a peak-to-peak deviation of 8.7° featuring marked extremes at four distinct chip inclinations, namely at ±30° and 180 ± 30° (see Figure 14). These directions have no counterpart in the symmetry of the transducer design. Hence, imperfections of the measurement setup must be responsible for observed deviation. 

To clarify the observed high-velocity effects, the 3D FEM model of the setup was evaluated for an inflow velocity of *U*_mean_ = 5 m/s. For the simulations, the channel orientation was fixed while the transducer assembly was rotated clockwise by an angle φ.

**Figure 15:**
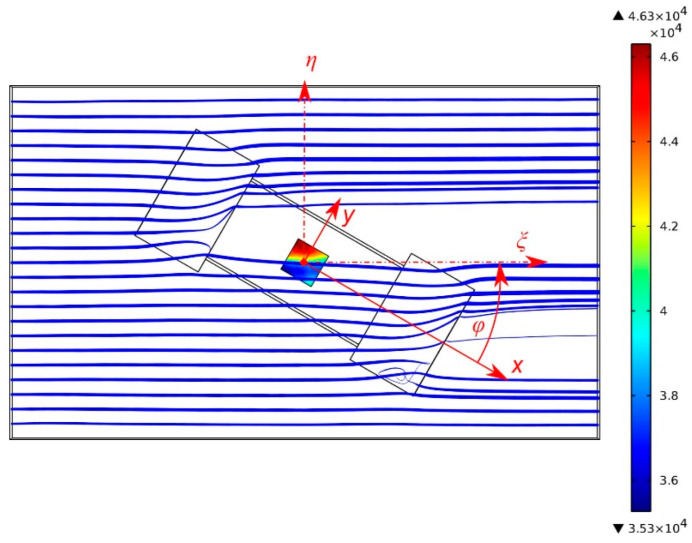
Streamlines (particle trajectories from left to right, 2D projection) starting from the left-hand side at a distance of 100 µm above the bottom channel wall, which incorporates the transducer chip as well as the lateral recesses. The color map quantifies the shear rate at the transducer membrane. The width of the streamlines illustrates the local flow magnitude. The image hides any streamline deformation perpendicular to the image plane. The mean inflow velocity amounts to 5 m/s, and the relative yaw of axes measures 30°.

Figure 15 displays a selected simulation result for a yaw angle of +30° with respect to the *x*-axis of the chip. It shows computed streamline tubes that originate 100 µm above the bottom channel wall. The width of these tubes scales with the velocity magnitude. As we deal with steady flows, the course of the streamlines also represents particle trajectories. 

Figure 15 suggests that the shown streamlines significantly penetrate into the interconnection recesses. Of course, they also bend slightly toward the chip surface, which is lowered by 50 µm with respect to the bottom channel wall. 

Only a moderate directional deviation from the channel axis occurs at the site of the transducer membrane. In contrast, a massive lateral deflection of these near-wall particle trajectories occurs at the larger lateral recesses. The closer the starting point to the bottom wall, the lower the inflow velocity of the respective streamlines and the more pronounced the lateral displacement. 

Reciprocally, the lateral distortion of the streamlines decreases for starting points with increasing distance from the membrane surface. Consequently, the direction of the fluid velocity depends on the distance from the duct walls in the neighborhood of discontinuities of that wall. Although the fluid volume is perfectly guided along the duct, there is no unique flow direction above a fixed position of the chip surface. The term “transducer deviation” in the presented diagrams refers the flow direction computed from the transducer bridge signals to the *ξ*-axis of the duct. 

**Figure 16:**
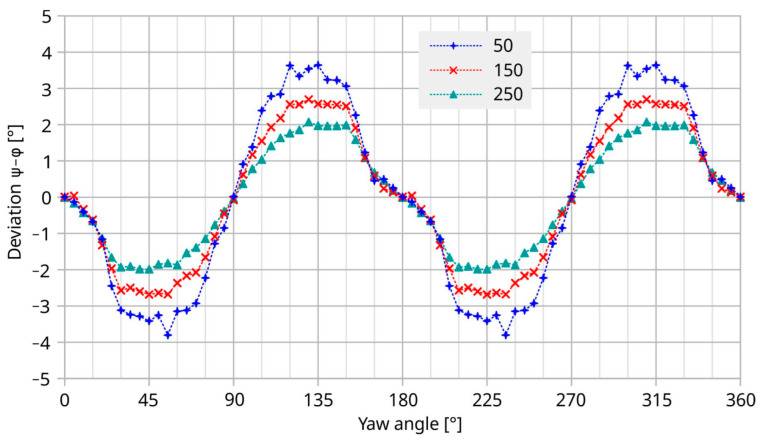
Simulated variation in the azimuth of the fluid flow velocity as a function of the yaw angle. The curves refer to the membrane center and distances of 50, 150, and 250 µm above the chip surface and *U*_mean_ = 5 m/s.

In contrast to the rapid change in the initial deviation of Figure 14, the moderate yaw of surface-near streamlines at the membrane location (see Figure 16) exhibits only a smooth and gradual change with the yaw angle of the transducer. Furthermore, the yaw of the tangential velocity vector decreases with increasing distance from the wafer surface. Therefore, the peaks of the transducer deviation of Figure 14 are not determined by these moderate changes in the flow direction by the discontinuities of the bottom channel wall.

Figure 16 confirms that streamlines starting more distant from the bottom channel wall are less deflected by surface discontinuities. To maintain fluid continuity, lateral displacement of surface-near trajectories requires that streamlines originating more distant from the bottom wall bend toward this wall. Moreover, they belong to higher velocities according to the specified inflow profile of Equation (6). As a consequence, the surface-normal gradient of the flow magnitude rises in such regions. Hence, streamline deformation is accompanied by a variation in the shear rate $\dot{\gamma }$ at the transducer surface

(7) $$\dot{\gamma }=\frac{\partial v_{\xi }}{\partial \eta }+\frac{\partial v_{\eta }}{\partial \xi }\approx \frac{\partial v_{\xi }}{\partial \eta }$$

Therefore, the surface-normal velocity gradient, i.e., the shear rate of Equation (7), is higher the more massive the lateral displacement of surface-near streamlines. 

Correct 2D calorimetric transduction of the intended flow direction requires a symmetric distribution of the membrane temperature with respect to the *ξ*-*ζ* plane, i.e., the symmetry plane of the duct. The same symmetry is required for the strength of convective cooling if the thermal behavior of the membrane features central symmetry. Convective cooling is tightly coupled to the local surface shear rate [30]. Therefore, the velocity field must also be symmetric to the *η* = 0 plane. 

Obviously, the surface shear rate distribution of Figure 15 violates this symmetry condition (see color-encoded membrane area). A marked variation in the shear rate across the intended flow direction occurs due to the asymmetric deformation of surface-near trajectories. Under these circumstances, the transducer output, Equation (3) cannot be interpreted in terms of flow directions. In the current setup, the symmetry of the flow velocity field is destroyed by the recesses of the inclined transducer mount. Even in the case of a perfect velocity field, a deviation may arise if the transducer is mounted asymmetrically within the duct.

The deformation of the streamlines as well as their distribution are also strongly dependent on the transducer yaw in the channel. A sequence of streamline pictures, such as that of Figure 15, taken in steps of 5° over the interval from zero to 90°, unveils that the distortions of the velocity field are most severe in four angular regions of the chip orientation where the deviation between the *x* and *ξ* axes ranges from 10 to 40°. The equivalent ranges are obtained by 180° rotation of the chip as well as by mirroring these positions at *η* = 0. This observation is in good agreement with the largest undulations of the initial deviation shown in Figure 14.

**Figure 17:**
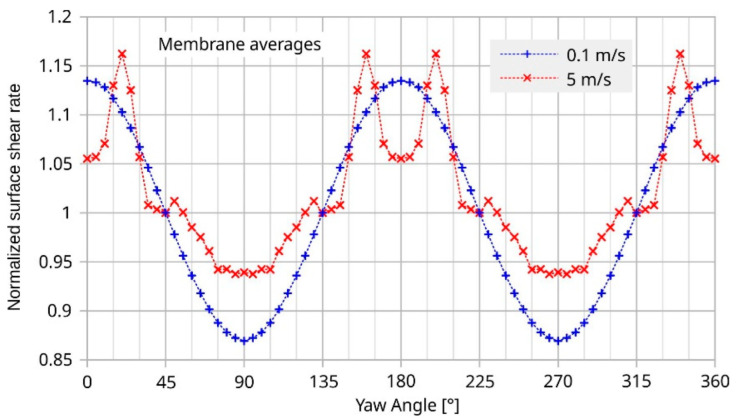
Simulated angular dependence of surface shear rates. The curves represent averages over the entire membrane area. They are normalized to their values at a yaw angle of 45°. The applied normalization constants are 659 s^−1^ and 39,847 s^−1^ for *U*_mean_ 0.1 m/s and 5 m/s, respectively.

Figure 17 highlights simulated angular dependencies of the surface shear when averaged across the membrane area. For *U*_mean_ = 0.1 m/s, the surface shear rate and the related magnitude conversion (see Figure 11) show similar angular dependencies. 

At 5 m/s, the common feature of the angular characteristics of transducer deviation and average surface shear rate are ostentatious variations in the same angular intervals (roughly −45 to +45 and 135 to 225°; compare Figure 14 and Figure 17). 

**Figure 18:**
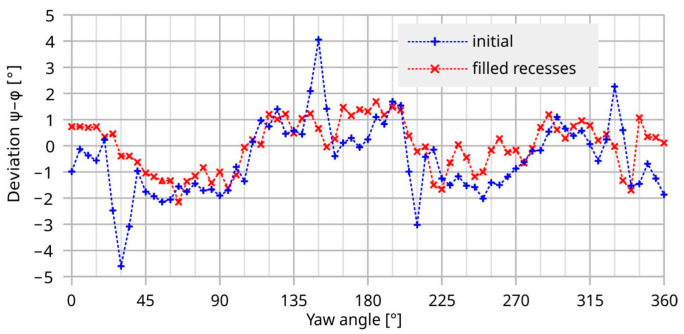
Improvement of the deviation by (partial) leveling of the recesses of the setup at a mean flow velocity 5 m/s.

Figure 17 highlights a rapid variation in the shear rate around 30° and the equivalent yaw angles. The initial deviation characteristic of the transducer shown in Figure 18 exhibits narrow extrema at the very same yaws. Hence, shear rate asymmetry seems to be the dominant mechanism leading to a deviation of the transducer output, while the height-dependent distortion of the flow direction seems less effective. The deviation of the measured flow direction (computed from *U*_BX_ and *U*_BY_ applying Equation (3)) from the preset yaw angle depends strongly on the magnitude of the flow velocity, even without any surface irregularities around the sensor membrane (see Figure 4, which shows simulation results assuming an ideal sensor, and Figure 5, which shows measurement results). On the other hand, the deviation of the measured flow magnitude (computed from *U*_BX_ and *U*_BY_ applying Equation (4) as well as the |*U*_B_| (*v*)-characteristic) from the preset flow magnitude |*v*| depends on the slope of *U*_B_ (*v*)-characteristic (see Figure 7). In the “saturation” region (i.e., *v* > 2 m/s), the slope is approximately equal to zero and the relative error is very low, as can be seen in Figure 13 (the nearly perfect circular shape of the directional diagram). In contrast, at 0.1 m/s, the directional diagram differ strongly from the circular shape (see Figure 8).

To summarize, membrane-based calorimetric flow transduction suffers from intrinsic deviation if the flow velocity field lacks symmetry with respect to the *η* = 0 plane. 

Substantial reduction in the observed peak-to-peak deviation is achieved by filling up the interconnection openings with glycerol. However, this measure yields no perfectly flat flow boundary due to the concave shape of the opening, i.e., the liquid is higher at the edge than in the center due to surface tension and wetting. According to Figure 18, a reduction in the peak-to-peak deviation from 8.7° to 3.8° is achieved. The latter figure comprises a further arbitrary error of about 1° that is introduced by the manual yaw angle setting. Therefore, the final measurement results are quite compatible with the related 3D simulations of Figure 4 that predict 1.7° peak-to-peak deviation for the bare transducer element.

Certain points of view on the impact of surface discontinuities on the transduction characteristics of a membrane-based thermal flow transducer have been investigated in [31], where the influence of different surface elevations of a 1D-transducer with respect to the flow channel wall has been studied. Alterations of unidirectional transduction characteristics were found that are in reasonable accordance with the presented results in [31]. 

In conclusion, even small surface discontinuities near the transducer membrane may cause marked distortions of the boundary layer of the flow. The higher the surface shear rate, the more severe these distortions. Furthermore, any lateral magnitude variation in the velocity at the membrane location transforms into irregular variations of the transducer signals, which in turn leads to erroneous interpretation of the flow direction. Then the transducer bridge signals are no longer representative of the tangential components of the inflow velocity.

## 4. Conclusions

Even small-sized, shallow surface irregularities evoke severe distortions of the tangential flow velocity around them. These effects are exaggerated with shrinking thickness of the boundary layer, i.e., with increasing surface-normal gradient of the magnitude of the tangential flow velocity. Membrane-based 2D flow transducers incorporated in a setup enabling 360° rotation of laminar gas flows can confirm these findings. In the current investigations, recesses required for electrical access to the membrane-borne resistive transducer elements served as surface discontinuities. Related FEM results showed that local changes in the velocity magnitude and direction occur. These flow field characteristics affect the output of the 2D-transducer in a very complex manner and prohibit direct interpretation of transducer signals.

For the sake of simplicity and clarity, we presented measurement only for the two extreme cases of very high slope (0.1 m/s) and very low slope (5 m/s) of the *U*_B_ (*v*)-characteristic. In the “transition region” (i.e., 0.5 m/s < *v* < 1 m/s), the flow direction deviation (which is the main topic of interest in our paper) is between the boundaries determined by the measured values for 0.1 m/s and 5 m/s.

With respect to the angular accuracy, FEM results predicted a peak-to-peak deviation of 1.7° for perfect flow boundaries, which is comparable with the 3.8° of the current experiment in spite of its shortcomings.

A distortion-free measurement of 2D flow fields has the following prerequisites. First, a perfectly flat interface to the fluid is required regarding the transducer chip itself as well its surrounding attachment. That makes through-wafer access to the membrane-borne thin-film resistors mandatory. Second, the transducer assembly should form a thin, perfectly circular disk with cuneiform periphery. Third, the disk diameter should be large enough to prevent eventual flow vortices from reaching the membrane region, i.e., to enable a flat transversal profile of the tangential flow velocity throughout the transducer membrane. A wide flow range demands constant-temperature operation, whereas a small disk diameter is preferable if high sensitivity at low wind speeds is needed.

## Figures and Tables

**Table 1:** Sensor dimensions (* overall length of a single U-shaped heater).

	Material	Thickness	Width	Length
Chip	Si	350 µm	3 mm	6 mm
Membrane	SiN_x_–SiO_2_–Si_3_N_4_	1.5 µm	1.2 mm	1.2 mm
Heater	Cr	0.27 µm	5 µm	1225 µm *
Thermistor	aGe	0.27 µm	Triangle sides	*a* = 530 µm *b* = *c* = 372 µm

## Data Availability

Data sharing is not applicable to this article.
